# Has urban public service equalization reduced regional differences in economic resilience?

**DOI:** 10.1371/journal.pone.0303236

**Published:** 2024-08-26

**Authors:** Susu Wang, Qidi Zhang, Mengze Sun, Yuhong Teng

**Affiliations:** 1 School of Business, Liaocheng University, Liaocheng City, China; 2 Shandong Academy of Social Sciences, Jinan, China; 3 Library of Zhongnan University of Economics and Law, Wuhan, China; 4 School of Business, Jianghan University, Wuhan, China; Chang’an University, CHINA

## Abstract

We delve into whether the equalization of basic public services can mitigate regional disparities in China’s economic resilience. Our analysis reveals that COVID-19 has diminished economic resilience and exacerbated regional differences. Notably, these regional disparities constitute the primary cause of spatial variations in economic resilience. Despite the initially low level of basic public services in Chinese cities, there is a discernible upward trend, indicating a gradual narrowing of regional disparities. Furthermore, we uncover a substantial positive correlation between the equalization of public services and variations in regional economic resilience, thereby offering fresh empirical evidence that the equalization of public services can help bridge the gap in regional economic resilience.

## 1. Introduction and literature review

Strong resilience is a key factor for China’s steady economic growth, a strong support for preventing risks, and an important guarantee for high-quality economic development. In the face of the impact of the COVID-19 epidemic, the Chinese economy has shown strong resilience by quickly returning to the previous growth path or reallocating resources to explore a new growth path in accordance with its self-recovery and adjustment functions. Economic resilience is the ability of an economic system to resist shocks and maintain its own structure and function, and the resilience of an economic system to cope with shocks through rapid diversification of response measures (Hassink, 2010; Oliva and Lazzeretti, 2018) [1, 2], mainly including four process dimensions of vulnerability, resistance to shocks, adaptation to shocks, and recovery from shocks (Martin et al., 2015) [3]. The equalization of basic public services can add new impetus to the formation of economic resilience through multiple adjustment mechanisms, such as promoting education equity, narrowing income gap, encouraging entrepreneurship and innovation, and ensuring basic infrastructure. In 2005, "equalization of public services" first appeared in the "Proposals on the formulation of the Eleventh Five-Year Plan" document, in 2018 issued and implemented the "Guiding Opinions on Establishing and improving the Standard System of Basic Public Services", in 2022, the report of the 20th National Congress of the Communist Party of China pointed out that "sound basic public services are vaguely, To improve the level of public services, enhance balance and accessibility, and solidly promote common prosperity, the equalization of public services has become one of the important contents of China’s economic development and the formation of economic resilience.

The idea that all residents of a region should enjoy equal access to basic public services comes from the concept of fiscal balance proposed by Buchanan (1950) [4]. Parity of public services means that all citizens have fair and accessible access to roughly equal basic public services, with its core being to promote equal opportunities, focusing on guaranteeing people’s access to basic public services, rather than simple averaging.

Haiyan County, as the only pilot county for the equalization of basic public services in Zhejiang Province, has been committed to promoting the balanced development of urban and rural areas in recent years with "urban-rural integration" as the carrier, actively carrying out exploration and practice in the fields of basic livelihood services and public utility services, and building a basic public service system covering urban and rural areas and sustainable development. In 2018, the per capita disposable income of rural residents in the county was 34,853 yuan, and the income ratio of urban and rural residents shrank from 1.99:1 before the equalization of basic public services reform to 1.70:1.

The regional equalization of basic public services belongs to the spatial equity of resource allocation. Basic public services are also a requirement for achieving regional economic development, rural revitalization and poverty eradication (Dittmar and Meisenzahl, 2020) [5]. Investments in public services, such as education and healthcare, have raised China’s human capital ranking from 69th in 1990 to 44th in 2016 (Lim et al., 2018) [6]. Due to the free nature of basic public services, a better provision of basic public services means a higher level of subjective well-being under the condition that the income level of rural households remains the same (Baldini et al., 2018) [7], but in rural areas, due to the serious problems of rural aging and hollowing, it is difficult to ensure the needs of left-behind children and the elderly for education, medical care and elderly care services (Pan et al., 2022) [8]. Although promoting the equalization of public services can help narrow regional disparities, there is significant heterogeneity in the impact of public services on regional income disparities due to the different preferences of local governments for different types of public service supply (Li et al., 2017) [9]. The degree of urbanization can greatly improve the level of equalization of public services (Cheng et al., 2024) [10], and the inequality of public services between regions widens the income gap between the eastern, central and western regions, leading to excessive labor transfer to the eastern region, reducing the efficiency of resource allocation and social stability, and hindering the common prosperity of all people (Patricia, 2022) [11].

As the "bottom line" of the government’s public functions, basic public services require the government to assume ultimate responsibility; As the core and important part of public services, basic public services are directly related to the interests of citizens, and are the minimum scope of maintenance of national economic and social stability, protection of individuals’ most basic rights to subsistence and development at a certain stage (Li et al., 2020) [12]. Therefore, the provision of institutional policies is of great significance to the equalization of public services. On the other hand, the equalization of public services can be related to the vital interests of the people, and the gap in basic economic services may affect social stability and economic development. The socialist market economic system must not only properly handle the relationship between the market and the government, but also handle the relationship between the government, the market, and society. Government is a booster of social and market progress; Society is the source of government and market dynamics; The market is a reflection of society and the object of government services, mainly to achieve the government’s promising, effective market, and social order. The relationship between the government and the market generally refers to the economic field, but whether it is reasonable and effective is largely reflected in whether the society is stable and harmonious, whether the fruits of development are shared, and whether the broadest masses of the people have a “sense of gain”, “security” and “sense of ownership”. If the social and economic achievements are not enjoyed by the people, social contradictions will intensify, which will lead to the “double failure” of the government and the market. Therefore, the equalization of basic public services and economic resilience play an important role.

The current research focuses on the relationship between the equalization of basic public services and the gap between regional economic resilience, which has not been studied extensively in existing literature. To address this gap, the study constructs a comprehensive index system of basic public services and measures the supply level of basic public services in 274 Chinese cities from 2011 to 2020 using the entropy weight method. The Dagum Gini coefficient and its decomposition method are used to analyze the spatial differences and sources of economic resilience and basic public services, while the social relationship data analysis paradigm is used to study the relationship between them. A relational data econometric model is also constructed to investigate the relationship between the equalization of basic public services and the regional difference in economic growth quality, using the quadratic assignment procedure. Our study provides empirical evidence to explore whether the equalization of basic public services can narrow regional differences in economic resilience, and introduces a new research paradigm for analyzing the causes of regional differences in economic resilience.

## 2. Research methodology

### 2.1. Indicator quantification method: Entropy evaluation method

In this paper, the entropy evaluation method is applied to evaluate the equalization of basic public services. The specific steps are as follows: *x*_*ij*_ denotes the value of the jth evaluation index in the ith year. *max*(*x*_*j*_) and *min*(*x*_*j*_) denote the maximum and minimum values of the jth evaluation index in all years, respectively. m is the number of evaluation years, n is the number of provinces. The specific calculation equation is as follows.


$$x_{ij}^{\prime }=\frac{x_{ij}-\min(x_{j})}{\max(x_{j})-\min(x_{j})}(Positive\;indicators)$$ (1)

$$x_{ij}^{\prime }=\frac{\max(x_{j})-x_{ij}}{\max(x_{j})-\min(x_{j})}(Negative\;indicators)$$ (2)

$$x_{ij}^{\prime }=\left\{ \begin{array}{c} \frac{x_{ij}-min(x_{j})}{x_{0}-min(x_{j})},x_{ij}<x_{0}\\ \frac{max(x_{j})-x_{ij}}{max(x_{j})-x_{0}},x_{ij}\geq x_{0} \end{array}\right.(Moderate\;indicators)$$ (3)

$$P_{ij}=\frac{X_{ij}^{\prime }}{\sum_{\alpha }\sum_{i}X_{ij}^{\prime }}$$ (4)

$$e_{j}=-k\sum_{i}P_{ij}\;\ln(P_{ij}),\;among\;themk=\frac{1}{\ln(mn)},\;and\;k>0,e_{j}\geq 0$$ (5)

$$d_{j}=1-e_{j}$$ (6)

$$W_{j}=\frac{d_{j}}{\sum_{j}d_{j}}$$ (7)

$$X_{ij}=x_{ij}^{\prime }\times W_{j}$$ (8)

### 2.2. Regional disparity measure: Dagum Gini coefficient and its decomposition

In this paper, we use the Dagum Gini coefficient decomposition method to study the regional differences in basic public services (economic resilience) and decompose the regional differences into three parts: intra-regional differences, inter-regional differences and the contribution of hypervariable density. The Gini coefficient is calculated with the Eq (9):

$$G=\frac{1}{2n^{2}\mu }\sum_{h=1}^{K}\sum_{j=1}^{K}\sum_{i=1}^{n_{j}}\sum_{r=1}^{n_{h}}\left|y_{ji}-y_{hr}\right|,$$ (9)

where the overall is divided into *K* groups (30 provinces in China are divided into two groups, i.e., the southern and northern regions), and *y*_*ji*_ and *y*_*hr*_ denote the basic public services (economic resilience) of any province (municipality and autonomous region) in *j*(*h*) regions, respectively, *j* = 1.2.…*K*, *h* = 1.2.…*K*. *G* denotes the overall Gini coefficient. *μ* is the average value of basic public services (economic resilience), *n* denotes the number of provinces, *n*_*j*_ and *n*_*h*_ are the number of regions within group *j*(*h*), respectively.

$$\mu_{h}\leq \ldots \mu_{j}\leq \ldots \mu_{K}$$ (10)

Following Dagum’s (1997) [13] decomposition of the Gini coefficient, the Gini coefficient *G* can be decomposed into three components: the contribution of intra-regional (within-group) disparities *G*_*w*_, the net contribution of inter-regional (between-group) disparities *G*_*nb*_ and inter-group hypervariance density *G*_*t*_. The latter two together measure the total contribution of inter-group inequality *G*_*gb*_ = *G*_*nb*_ + *G*_*t*_. The relationship between the three satisfies *G* = *G*_*w*_+*G*_*nb*_+*G*_*t*_. The intra-regional Gini coefficient *G*_*jj*_ and the contribution of intra-regional disparities *G*_*w*_, the inter-regional Gini coefficient *G*_*jh*_ and the contribution of inter-regional disparities *G*_*nb*_, and the hyper-variance density *G*_*t*_ are expressed as:

$$G_{jj}=\frac{1}{2{n_{j}}^{2}\mu_{j}}\sum_{i=1}^{n_{j}}\sum_{r=1}^{n_{j}}\left|y_{ji}-y_{jr}\right|$$ (11)

$$G_{w}=\sum_{i=1}^{K}p_{j}s_{j}G_{jj}$$ (12)

$$G_{jh}=\frac{1}{n_{j}n_{h}(\mu_{h}+\mu_{j})}\sum_{i=1}^{n_{j}}\sum_{r=1}^{n_{h}}\left|y_{ji}-y_{hr}\right|$$ (13)

$$G_{nb}=\sum_{j=2}^{K}\sum_{h=1}^{i-1}\left(p_{j}s_{h}+p_{h}s_{j}\right)G_{jh}D_{jh}$$ (14)

$$G_{t}=\sum_{j=2}^{K}\sum_{h=1}^{j-1}\left(p_{j}s_{h}+p_{h}s_{j}\right)G_{jh}(1-D_{jh}),$$ (15)

where $p_{j}=n_{j}/n,s_{j}=\frac{n_{j}\mu_{j}}{n\mu }.{\;G}_{jh}$ denotes the difference in basic public services (economic resilience) between *j*,*h* regions. When *μ*_*j*_>*μ*_*h*_,*d*_*jh*_ is the weighted average of the basic public service gap (economic resilience) in all regions under the condition of *y*_*ji*_>*y*_*hr*_. For continuous density distribution function *f*_*h*_(*y*) and *f*_*j*_(*y*), *d*_*jh*_ can be expressed as Eq (16). And *p*_*jh*_ is the hypervariable first-order moment, which can be interpreted as the weighted average of the basic public service gap (economic resilience) under the condition of *y*_*hr*_−*y*_*ji*_ when *μ*_*j*_>*μ*_*h*_, expressed by Eq (17).


$$d_{jh}=\int_{0}^{\infty }\int_{0}^{y}(y-x)f_{h}(x)dxf_{j}(y)dy$$ (16)

$$p_{jh}=\int_{0}^{\infty }\int_{0}^{y}(y-x)f_{j}(x)dxf_{h}(y)dy$$ (17)

### 2.3. Inspection method for formation mechanism of regional differences: Quadratic Assignment Procedure (QAP)

Regional differences in economic resilience are driven by differences in the development of factors such as equalization of basic public services, marketization, rationalization of industrial structure, openness to the outside world, and balance of deposits and loans of financial institutions in each region. Assuming region A and region B, whose economic resilience is denoted by *y*_*A*_ and *y*_*B*_, respectively, the difference in economic resilience between the two regions can be expressed as *y*_*A*_−*y*_*B*_. Supposing that the equalization of basic public services, marketization, rationalization of industrial structure, openness to the outside world, and balance of deposits and loans of financial institutions in region A(B) are denoted as $x_{A(B)}^{1},\;x_{A(B)}^{2},\;x_{A(B)}^{3},\;x_{A(B)}^{4},\;x_{A(B)}^{5}$. The differences in the five dimensions between region A and B can be expressed as $x_{A}^{1}-x_{B}^{1},\;x_{A}^{2}-x_{B}^{2},\;x_{A}^{3}-x_{B}^{3},\;x_{A}^{4}-x_{B}^{4}$ and $x_{A}^{5}-x_{B}^{5}$, respectively. Therefore, the difference in economic resilience between region A and B can be explained by the development difference of five factors.

The differences in economic resilience and development of various factors between the two regions can form multiple matrices. Regional differences in economic resilience (explained variables) and regional differences in various factors (explanatory variables) are set as relational data econometric models. With QAP analysis method, the formation mechanism of economic resilience differences is explored from the perspective of structural difference relationship data.

Set the relational data measurement model as shown in Eq (18), where *β*_0_,*β*_1_⋯*β*_3_ are the parameters to be estimated. *X* and *Y* are the explanatory variables (regional differences in each factor) and the explanatory variables (regional differences in economic resilience), respectively. *U* is the residual term.

$$Y=\beta_{0}+\beta_{1}X_{1}+\beta_{2}X_{2}+\beta_{3}X_{3}+U$$ (18)

The difference between relational data measurement model and general data measurement model lies in that the variables in the model are in the form of n-order square matrix, as shown in Eq (19), where *y*_*ij*_ (*i* = 1⋯*n*,*j* = 1⋯*n*) represents the difference in economic resilience between regions *i* and *j*. $x_{ij}^{m}$ represents the *m*(*m* = 1⋯3) dimensional difference of economic resilience between *i* and *j*, which is calculated by $y_{ij}=y_{i}-y_{j},\;x_{ij}^{m}=x_{i}^{m}-x_{j}^{m}$, respectively. When *i* = *j*, the diagonal of the square matrix is 0.

$$Y=\left(\begin{array}{ccccc} 0 & y_{12} & \cdots & y_{1(n-1)} & y_{1n}\\ y_{21} & 0 & \cdots & y_{2(n-1)} & y_{2n}\\ \vdots & \vdots & \ddots & \vdots & \vdots \\ y_{(n-1)1} & y_{(n-1)2} & \cdots & 0 & y_{\left(n-1)(n-1\right)}\\ y_{n1} & y_{n2} & \cdots & y_{n(n-1)} & 0 \end{array}\right)$$

$$X_{m}=\left(\begin{array}{ccccc} 0 & x_{12}^{m} & \cdots & x_{1(n-1)}^{m} & x_{1n}^{m}\\ x_{21}^{m} & 0 & \cdots & x_{2(n-1)}^{m} & x_{2n}^{m}\\ \vdots & \vdots & \ddots & \vdots & \vdots \\ x_{(n-1)1}^{m} & x_{(n-1)2}^{m} & \cdots & 0 & x_{\left(n-1)(n-1\right)}^{m}\\ x_{n1}^{m} & x_{n2}^{m} & \cdots & x_{n(n-1)}^{m} & 0 \end{array}\right)$$ (19)

The row and column elements in the residual matrix U in the relational data econometric model are not independent. Moreover, there is serious multicollinearity problem between variables whose observations are relational data. The parameter estimates and standard deviations obtained by traditional statistical test methods are increased, which leads to the loss of significance of the significance test of variables, and is not applicable to the traditional statistical methods. Quadratic Assignment Procedure (QAP) is a hypothesis testing method for two types of relationships, which does not require the assumption that the variables are independent of each other and could effectively avoid autocorrelation and multicollinearity problems in the data measurement model. This method is more suitable for the study on the formation mechanism of regional differences in economic resilience. QAP includes correlation analysis and regression analysis. QAP correlation analysis examines the correlation between variable matrices, and QAP regression analysis examines the regression relationship between the explanatory variables (*X*_*m*_ matrix) and the explanatory variables (*Y* matrix). The principles of the two are basically the same, including two steps. Take QAP regression analysis as an example.

Step 1: long vector regression. The relationship matrix is converted into an *n*×(*n*−1) dimensional column vector, i.e., a long vector. OLS estimation is performed to calculate the regression coefficients.

$$Y=\left(\begin{array}{c} y_{12}\\ y_{13}\\ \vdots \\ y_{n(n-1)} \end{array}\right),\;X_{m}=\left(\begin{array}{c} x_{12}^{m}\\ x_{13}^{m}\\ \vdots \\ x_{n(n-1)}^{m} \end{array}\right)$$ (20)

Step 2: Random permutation and statistical test. After conducting the multivariate QAP regression, the DSP method, which is more robust in the residual matrix replacement method, is chosen to conduct the statistical test. Assuming that *t* random permutations are performed, where the number of times greater than or equal to, less than or equal to the long vector regression coefficients in the first step are denoted as *t*_*l*_ and *t*_*s*_, respectively. Then we obtain two ratios p1(*p*_*l*_ = *t*_*l*_/*t*) and *p*_*s*_(*p*_*s*_ = *t*_*s*_/*t*), which can be directly regarded as the minimum significance level for rejecting the original hypothesis, i.e., the p-value of the statistical test. The regression coefficients are used as a two-tailed test, which will be used as the P-value of the statistical test if the regression coefficient is positive, and as the p-value of the statistical test if the regression coefficient is negative.

## 3. Data description and statistical analysis

### 3.1. Data description

Urban public services. Basic public services include three basic parts: first, to guarantee the basic right of human existence; second, to meet the needs of basic dignity and basic ability; third, to meet the needs of basic health. As far as the current stage of economic development is concerned, compulsory education, public health and medical care, basic social security, and public employment services are the most concerned and urgent public services for the majority of urban and rural residents, becoming the main content of basic public services in China at this stage. The evaluation index system of basic public service capacity is divided into subjective evaluation and objective evaluation. Based on the “Notice on Promoting the Equalization of Basic Public Services” of the 12th and 13th Five Year Plan of China, this paper mainly examines the financial input made by local governments in basic public services and the objective results achieved from the perspective of objective evaluation. This paper constructs a comprehensive indicator evaluation system of basic public services based on social security, health care, education, infrastructure and employment. The entropy evaluation method is used to measure the level of basic public service provision (PS) in 274 cities using city-level data from 2011 to 2020.The content of the index system is described in Table 1.

**Table 1:** Comprehensive index system of urban basic public services.

Level of urban public service provision (2011–2020)	Social Security	Number of urban workers’ pension insurance participants/number of employed population	Direction of action
		Number of unemployment insurance participants/number of employed persons	Positive
		Number of urban workers’ basic medical insurance participants/number of employed persons	Positive
	Health Care	Number of beds in medical institutions/total population	Positive
		Number of doctors/total population	Positive
	Education	Elementary school teacher-student ratio	Positive
		Secondary School Teacher-to-Student Ratio	Positive
		Financial education expenditure/fiscal expenditure	Positive
	Infrastructure	Public library book collections per person	Positive
		Number of buses/total population	Positive
		Taxis/total population	Positive
		Year-end actual road area/total population	Positive
	Employment	Wage levels of employees by city/wage levels of employees nationwide	Positive
		Number of employees/population per unit at the end of the year	Positive

Economic resilience. In this paper, we refer to the sensitivity index *SI* commonly used by Martin (2012) [14], Faggian et al. (2018) [15] to measure economic resilience (*ER*).The specific formula is *S*_*it*_ = (*lnL*_*it*_−*lnL*_*it−k*_)−(*lnL*_*t*_−*lnL*_*t−k*_), where *lnL*_*it*_ is the number of employees in province *i* in year *t*, *lnL*_*t*_ is the number of employed people in the whole country in year *t*, and the subscript *t*−*k* indicates *k* years ago. In this paper, we assume that *k* = 1. The economic resilience index is constructed by the above method. If *S*_*it*_ is greater than zero, it means that the employment in province *i* grows faster (or declines slower) between *t*−1 and *t* years compared with the whole country, and its resilience is relatively strong; on the contrary, if *S*_*it*_ is less than zero, it means that employment in province *i* grows more slowly (or declines more rapidly) between *t*−1 and *t* years compared to the whole country, and its economy is relatively less resilient.

Control variables. The marketization index(MI) is derived by Fan et al. (2003) [16], which consists of the relationship between government and market, the development of the non-state economy, product markets, factor markets, market intermediary organizations, and the legal environment of the market, matched to the city level using the provincial index. Trade is measured by urban trade import and export. The rational structure of production (RSP) is calculated as: (*gdp*_*it*_/*gdp*_*t*_)*ln((*L*_*i*_/*L*_*t*_)/(*gdp*_*i*_/*gdp*_*t*_)), where *i* takes the values of 1, 2, 3, representing primary, secondary and tertiary industries, and *t* represents time and takes the values of 2011–2020. DI of financial institutions is measured by the deposit and loan balance of urban banking financial institutions.

### 3.2. Statistical analysis

#### 3.2.1. Economic resilience

**Fig 1:**
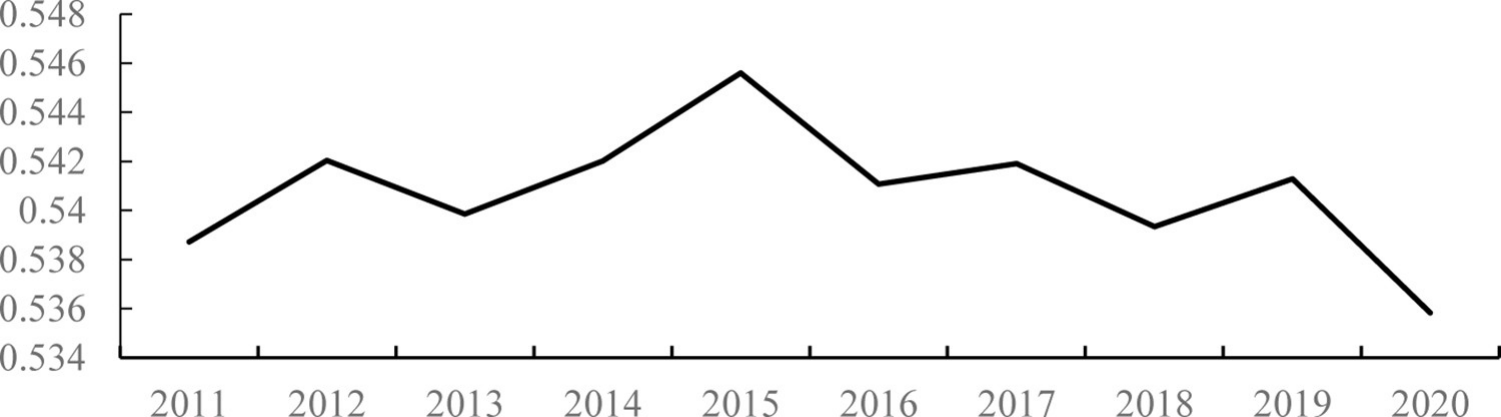
Trends in the economic resilience of cities in China (2011–2020).

As shown in Fig 1, during the sample period from 2011 to 2020, the economic resilience of Chinese cities exhibited a “rising-declining” trend. It rose from 0.539 in 2011 to a maximum value of 0.546 in 2015, and then showed a fluctuating decline trend since 2015, falling to 0.536 by 2020, with a decline rate of 0.54%. The smaller decline in urban economic resilience in recent years is mainly due to the impact of the US-China trade war in 2019 and the COVID-19 epidemic. For example, China suffered from COVID-19 in 2020, with economic growth rates of -6.8% in the first quarter of 2020, 3.2% in the second quarter, 4.9% in the third quarter, 6.5% in the fourth quarter, and an annual growth rate of 2.3%. Although China was the only country in the world to maintain growth, domestic economic development has been severely affected. Regarding employment, the proportion of employed persons to the population decreased from 56.5% in 2011 to 53.2% in 2020, and the number of employed persons also declined from 761.96 million in 2011 to 750.64 million in 2020.

**Fig 2:**
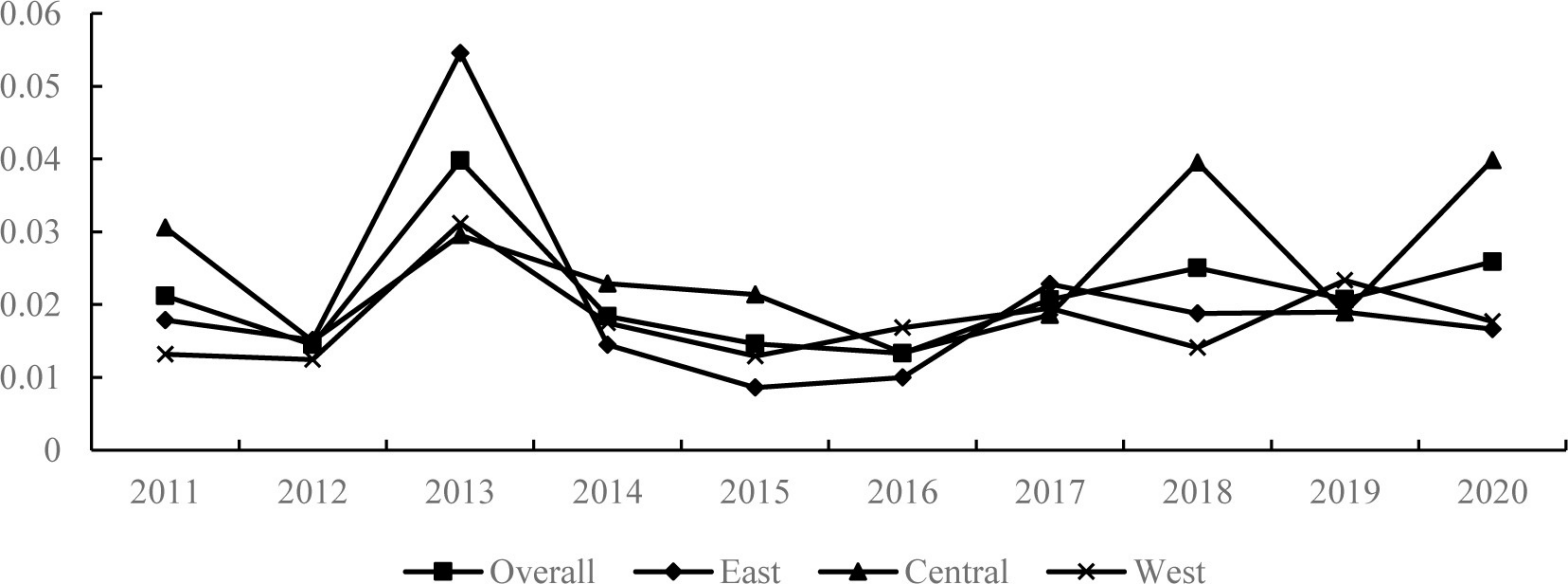
Evolution of the Gini coefficient of economic resilience.

The Gini coefficients of economic resilience of Chinese cities are measured and presented in Fig 2, both in general and by region. As shown in Fig 2, the Gini coefficient of economic resilience in China exhibits a small upward trend with fluctuations. Specifically, the Gini coefficient of economic resilience in the eastern region shows a decreasing trend, while the Gini coefficient in the central and western regions shows an increasing trend. From 2011 to 2013, the Gini coefficient of economic resilience of Chinese cities showed a small upward trend, rising from 0.021 in 2011 to 0.040 in 2013, indicating that regional differences in economic resilience of cities further expanded during that period. From 2014 to 2016, the Gini coefficient of economic resilience declined, falling from 0.040 in 2013 to 0.013 in 2016, indicating that regional differences in economic resilience narrowed during that period. From 2017 to 2020, the Gini coefficient of economic resilience showed a small growth trend and overall showed a trend of expanding regional differences. The overall Gini coefficient of the eastern region shows a decreasing trend, indicating that regional differences in economic resilience in the eastern region have narrowed during 2011–2020, and cities have developed in a coordinated manner. The Gini coefficient of economic resilience in the central region shows a “decreasing-rising” trend in general, and the overall performance shows the expansion of regional differences. The Gini coefficient of economic resilience in the western region exhibits a fluctuation trend similar to that of the whole country, with an overall trend of growth and further expansion of regional differences in economic resilience. Taking 2008–2020 as an economic cycle, 2008–2010 is in the period of economic expansion, 2011–2015 is in the period of economic adjustment, and 2016~2020 is in the period of economic contraction. During the period of economic adjustment and economic contraction from 2011 to 2020, the improvement of the resistance resilience of the eastern region was mainly due to the enhancement of "quality", while the "qualitative" resistance resilience of the central and western regions was weaker than that of the eastern region, which narrowed the difference in economic resilience in eastern China and widened the difference in economic resilience in the central and western regions.

**Fig 3:**
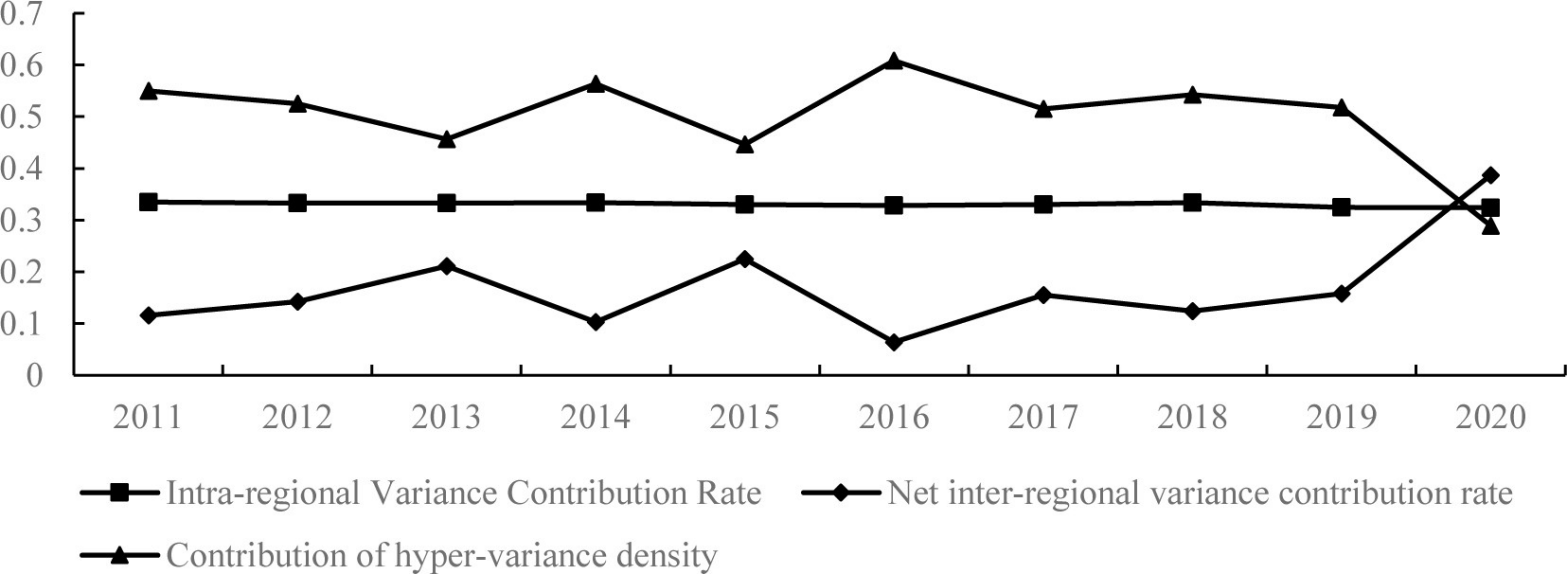
Trends in the sources of spatial variation in China’s economic resilience.

The trends of the sources of spatial variation in China’s economic resilience are demonstrated in Fig 3. From Fig 3, it can be observed that the inter-regional variance contribution rate, which consists of the net inter-regional variance contribution rate and the hyper-variance density contribution rate, is the primary source of spatial variance in economic resilience of Chinese cities, with a contribution rate ranging from 66.52% to 67.60%, and an average contribution rate of 66.95%. The intra-regional variance contribution rate is also an important source of spatial variance in economic resilience of Chinese cities, with a contribution rate ranging from 32.40% to 33.48%, and an average contribution rate of 33.05%. Among the inter-regional differences, the contribution of hyper-variance density is much higher than the contribution rate of net hyper-variance, which is the main source of inter-regional differences and also the main source of spatial differences in economic resilience of Chinese cities. However, as of 2020, the inter-regional net hyper-variance contribution shows a growing trend, increasing from 11.59% in 2011 to 38.68% in 2020, while the inter-regional hyper-variance density contribution shows a decreasing trend, falling from 54.93% to 28.92% in 2011. This indicates that the net difference problem between different regions has an increasing impact on the spatial differences of economic resilience, and the overlap problem between different regions has improved.

The main reasons for the differences in China’s economic resilience are as follows: the different resource endowments in the eastern, central and western regions lead to different levels of economic resilience. Compared with the central and western regions, the eastern region has higher resistance, resilience and innovation than the central and western regions due to its advantages of larger economic scale, relatively large population, more reasonable industrial structure, relatively high level of innovation and higher level of opening up, making the economic fluctuations of the eastern provinces smaller than those of the central and western provinces, and the economic resilience is stronger.

#### 3.2.2. The level of provision of basic public services

**Fig 4:**
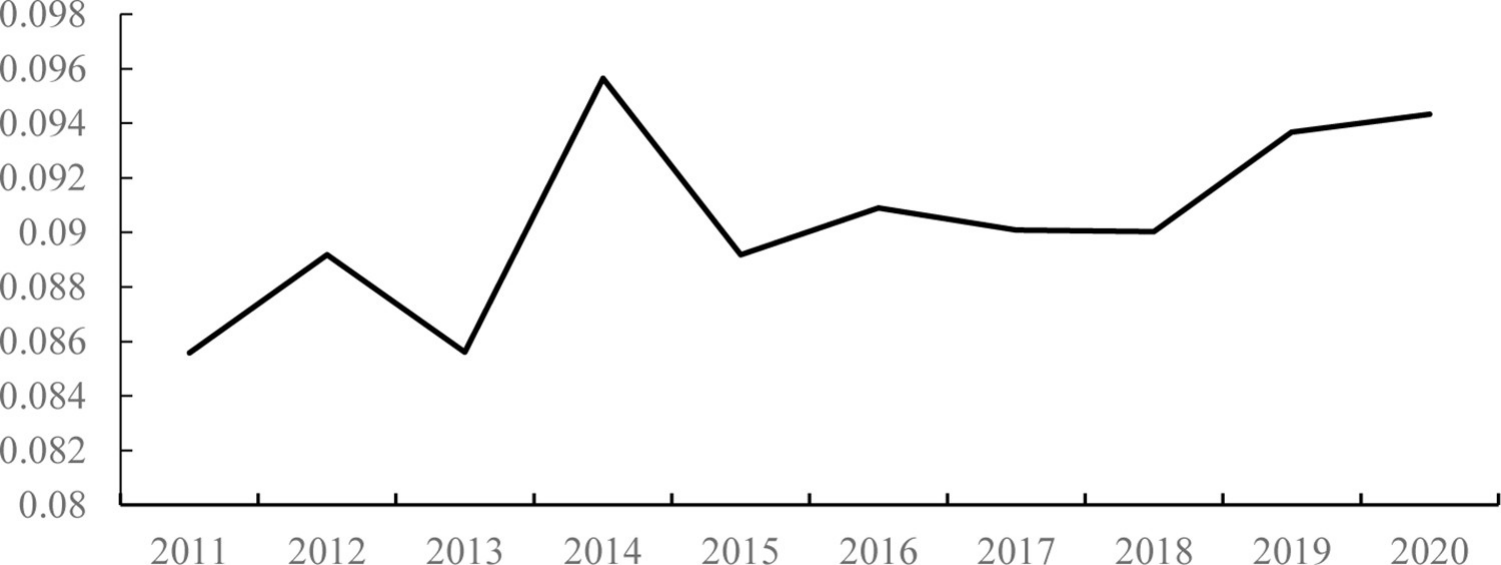
Trends in the level of public service supply.

The trend of urban public service supply in China from 2012 to 2020 is shown in Fig 4. As shown in Fig 4, the level of urban public service supply in China is generally low, with a public service supply index ranging from 0.086 to 0.096. However, there is a growth trend, with a growth rate of 10.22%, indicating that the level of urban public service supply in China has improved during the sample period. In recent years, China has made remarkable progress in medical and health care, basic society, and employment. As a result, medical and health conditions have significantly improved, infrastructure has gradually improved, and the employment system has gradually been established, leading to an increase in the level of public service provision. After calculation, the medical and health index increased from 0.135 in 2011 to 0.190 in 2020; the infrastructure improvement index increased from 0.058 in 2011 to 0.070 in 2020; and the employment index increased from 0.108 in 2011 to 0.109 in 2020, showing a small increase. However, the impact of the COVID-19 pandemic in 2020 has caused the above indices to shrink compared with 2019.

**Fig 5:**
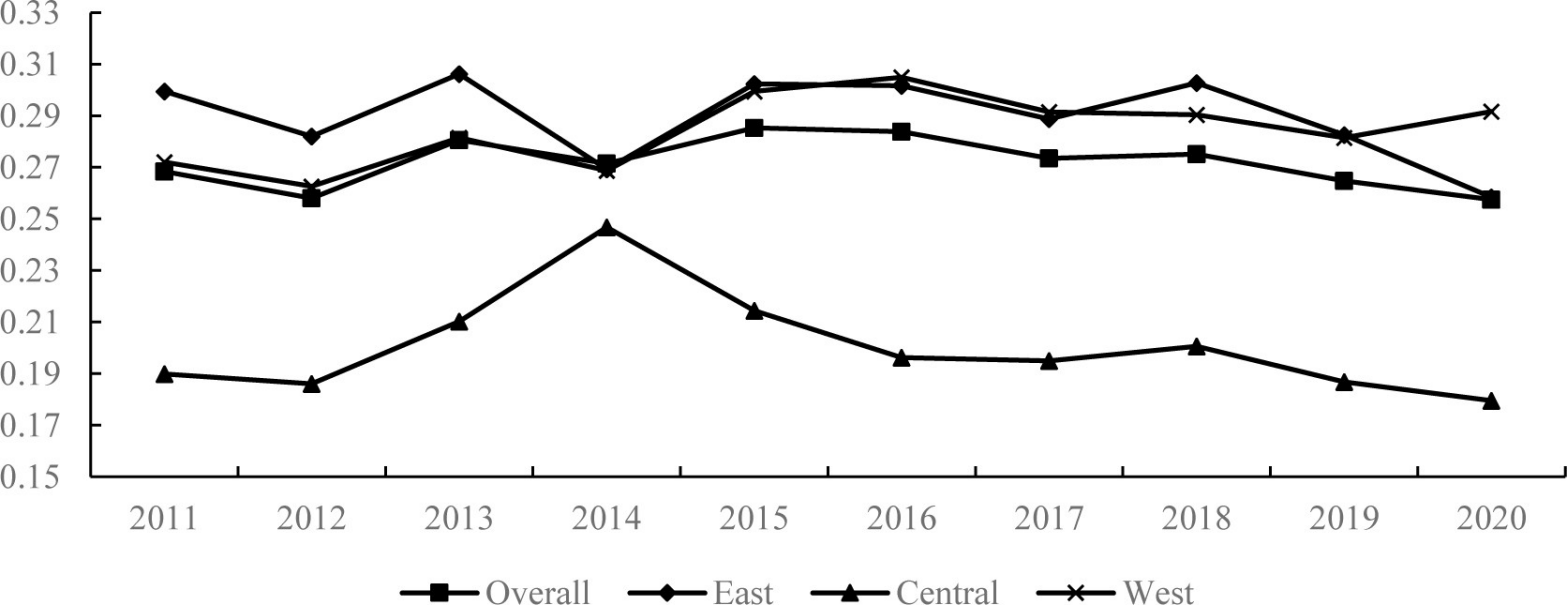
Changes in the Gini coefficient of basic public services in China.

In order to clearly understand the trend of equalization of basic public services, Fig 5 illustrates the change trend of the Gini coefficient of basic public services in China from 2011 to 2020. As shown in Fig 5, the Gini coefficient of basic public services in China has exhibited a significant decline overall, indicating that the Gini coefficient of urban public services in China has gradually become more equal. At the national level, the Gini coefficient of basic public services decreased from 0.268 in 2011 to 0.258 in 2020, indicating that basic public services have become more equalized. At the regional level, public services in the eastern and central regions showed a trend of equalization, and the Gini coefficient decreased to varying degrees during the period 2011–2020. The Gini coefficient of public services in the eastern region decreased from 0.300 in 2011 to 0.258 in 2020, and the Gini coefficient of public services in the central region decreased from 0.190 in 2011 to 0.180 in 2020. However, the regional difference between basic public services in the western region has further expanded, with the Gini coefficient increasing from 0.272 in 2011 to 0.292 in 2020.

The main reasons for the gradual equalization of basic urban public services are: First, the regional coordinated development strategy implemented by the state has brought positive results. Second, local governments have gradually changed their governing philosophy to a "public service-oriented" government; Third, we should implement industrial policies that are compatible with local resource endowments to achieve sound and sustainable economic development.

**Fig 6:**
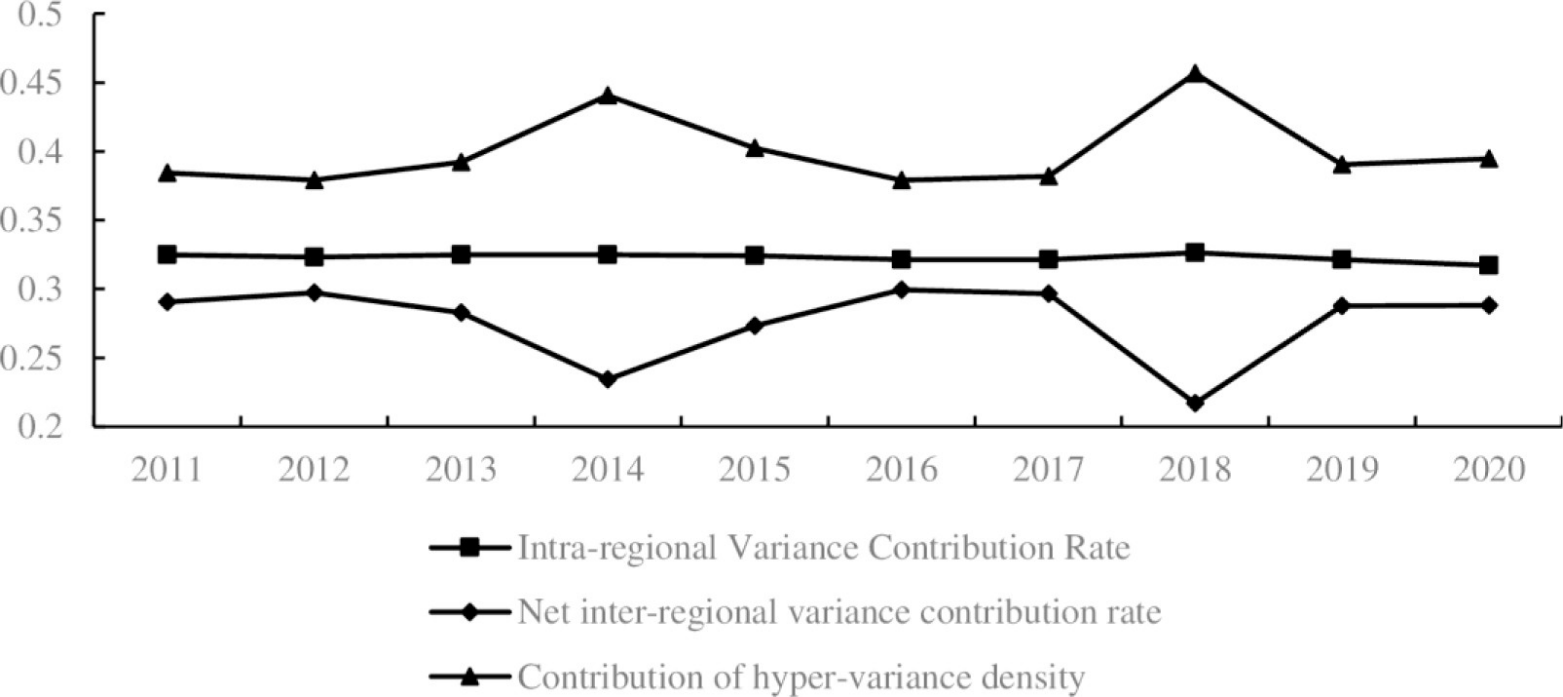
The dynamic trend of the spacial differences in basic public services in China.

The spatial differences of basic public services in China are derived from regional differences and interregional differences, with interregional differences further divided into interregional net value differences and interregional supervariable density. Based on Fig 6, the inter-regional difference is the main source of spatial difference in basic public services in Chinese cities, with a contribution rate ranging between 67.36% and 68.28%, and an average contribution rate of 67.7%, showing an increasing trend from 67.50% in 2011 to 68.28% in 2020, indicating that the contribution rate of interregional differences is increasing. Among them, the contribution rate of supervariable density between regions was much higher than that of inter-regional supervariable net value and intra-regional difference contribution rate, indicating that the overlapping problem of basic public service areas in Chinese cities is more severe. The contribution rate ranged from 31.72% to 32.64%, and the average contribution rate was 32.2%. However, the intraregional contribution rate showed a slight downward trend, from 32.50% in 2011 to 31.72% in 2020.

To summarize, there are regional differences in the economic resilience of Chinese cities, and the change trend is heterogeneous in time and region, with regional differences showing a trend of expansion during the sample period. Additionally, basic public services in Chinese cities are becoming more equalized, and spatial differences have been further narrowed. However, the Gini coefficient calculation is affected by individual extreme values and may not accurately observe the correspondence between samples. QAP focuses on the comparison between individuals from the perspective of relationship time. Therefore, to explore the impact of equalization of basic public services on differences in economic resilience and examine whether equalization of basic public services can reduce regional differences in economic resilience, the secondary assignment procedure is used to empirically test the correlation between the two.

## 4. Empirical testing

### 4.1. QAP correlation analysis

Table 2 presents the correlation analysis results of QAP during the observation period, and significant correlations were found between regional differences in economic resilience and regional differences in variables such as equalization of basic public services, marketization, rationalization of industrial structure, balance of deposits and loans of financial institutions, and opening up to the outside world. The difference in economic resilience and the difference in industrial structure rationalization showed a negative correlation, while the other indicators showed a positive correlation. Among these effects, the correlation between the difference in equalization of public services and the difference in economic resilience was second to the correlation with the difference in opening up (0.248), reaching 0.200. Other factors were closely related to regional differences in economic resilience, in descending order: deposit and loan balance of financial institutions (0.200), marketization (0.108), and industrial structure rationalization (-0.094). The above results indicate that the correlation between the variables, including the equalization of basic public services, and regional differences in economic resilience is significant. However, unlike the regression relationship, although the correlation between basic public services and economic resilience is close, it does not necessarily mean that basic public services have a greater impact on regional differences in economic resilience, and QAP analysis is required to test this.

**Table 2:** QAP correlation analysis results.

	ER	MI	RSP	DI	Trade	PS
ER	1***	0.108**	-0.094**	0.200***	0.248***	0.200***
MI	0.108**	1***	-0.037	0.093*	0.043	-0.056
RSP	-0.094**	-0.037	1***	-0.073***	-0.058	-0.010
DI	0.200***	0.093*	-0.073***	1***	0.869***	0.457***
Trade	0.248***	0.043	-0.058***	0.869***	1***	0.558***
PS	0.200***	-0.056	-0.010	0.457***	0.558***	1***

Furthermore, the results of Table 1 also indicate that there are significant positive correlations between economic resilience, deposit and loan balances of financial institutions, and regional differences in opening up to the outside world, and the equalization of basic public services, with correlation coefficients of 0.200, 0.457, and 0.558, respectively, passing the significance level test. However, given the general significant correlation between explanatory variables, there may be multicollinearity issues between explanatory variables, requiring QAP regression analysis to reveal the strength of the difference in economic resilience.

### 4.2. QAP full-sample regression

Table 3 presents the full-sample regression results where the explanatory variable is the difference in economic resilience. Based on the standardized regression coefficients in Table 3, the variables such as equalization of basic public services, opening up to the outside world, and marketization have significantly positive coefficients. This indicates that the equalization of basic public services, opening to the outside world, and the improvement of imbalance between market-oriented regions contribute to the narrowing of regional differences in economic resilience. On the other hand, the normalized regression coefficients of variables such as industrial structure rationalization and deposit and loan balances of financial institutions are negative but still statistically significant, suggesting that the rationalization of industrial structure and the narrowing of regional differences in deposit and loan balances of financial institutions may lead to further widening of the economic resilience gap. In terms of the size of the standardized regression coefficient, opening up has the highest impact on reducing regional differences in China’s economic resilience, followed by marketization and the equalization of basic public services. This indicates that the equalization of basic public services has played an important role in narrowing regional differences in China’s economic resilience, although its impact is slightly lower than that of opening up and marketization. The main reasons are: first, the equalization of basic public services can reduce the inequality of employment and medical opportunities, provide basic public facilities and basic social security, promote social fairness and justice, and make the economy socially resilient; Second, the equalization of basic public services can promote the upward income mobility of low-income people, expand the middle-income group, and make the economy scalable. Third, the equalization of basic public services can provide superior educational resources, making the economy innovative and resilient.

**Table 3:** QAP regulation analysis results.

Model variables	Unstandardized coefficients	Normalization factor	p-value	P_Large_	P_Small_
intercept	0.0000	0.0000	--	--	--
PS	0.0146	0.1008	0.0760	0.0760	0.9240
RSP	-0.0005	-0.0808	0.0570	0.9430	0.0570
Trade	0.0060	0.2558	0.0240	0.0240	0.9770
MI	0.0050	0.1078	0.0320	0.0320	0.9690
DI	-0.0008	-0.0845	0.2330	0.7680	0.2330

### 4.3. Robustness test

To verify the robustness of the narrowing effect of the gap between the equalization of basic public services and economic resilience, this study also conducted a regression analysis using GDP per capita as a substitute for economic resilience. Per capita GDP is not only an important indicator of economic development but also an indicator of the economic development trend. Economic resilience is manifested in the stability of the economy after external shocks or adverse economic conditions, and its ability to recover and achieve higher growth rates in the steady state. Therefore, GDP per capita can be considered an approximate substitute for economic resilience. Table 4 reports the impact of basic public equalization on the resilience gap using GDP per capita, which indicates that the equalization of basic public services narrows regional disparities in GDP per capita, with a standardized regression coefficient of 0.597 and a statistical significance level of 1%

**Table 4:** Robustness test.

Model variables	Unstandardized coefficients	Normalization factor	p-value	P_Large_	P_Small_
intercept	0.0000	0.0000	--	--	--
PS	34.573	0.5972	0.0000	0.0000	1.0000
RSP	0.0075	0.0030	0.3920	0.3920	0.6080
Trade	-0.4718	-0.0503	0.2900	0.7100	0.2900
MI	0.1449	0.0811	0.0230	0.0230	0.9770
DI	1.0390	0.2876	0.0010	0.0010	0.9990

## 5. Conclusions and policy recommendations

Existing literature has limited research on the relationship between equalization of basic public services and regional gaps in economic resilience from a relational perspective. This study introduces a relational data analysis approach to examine the gap between public service equalization and economic resilience. Using data from 274 cities in China between 2011 and 2020, the study constructs a basic public service supply level based on social security, healthcare, education, infrastructure, and employment. The study utilizes the secondary assignment procedure to analyze the relationship between public service and economic resilience regional gaps, which provides new empirical evidence to explore whether the equalization of basic public services can narrow the regional differences in economic resilience. The main findings of the study are as follows:

First, due to the impact of the COVID-19 pandemic, Chinese cities experienced a downward trend in economic resilience, and regional disparities have been widening. Regional differences were found to be the main source of spatial differences in urban economic resilience. The Gini coefficient calculation results revealed an increase in the gap between the economic resilience of Chinese cities, with a relative manifestation of "the stronger the stronger, the weaker the weaker". This indicates that more resilient cities in China perform better after adverse shocks, while less resilient cities perform worse after adverse shocks, and the difference between them is increasing. The main source of spatial differences in economic resilience is the supervariable net value between regions, indicating that cross-overlap between regions is a serious issue and the main cause of spatial differences.

Second, although the overall level of public service supply in China’s cities remains low, there is a positive trend in its growth and regional differences are gradually narrowing. This trend is a result of significant progress made in areas such as medical and health, basic society, and employment, which has led to improvements in medical and health conditions, infrastructure, and employment systems, thereby contributing to higher levels of public service provision. Additionally, the Gini coefficient of urban public services in China has been gradually equalizing. Inter-regional differences are the primary source of spatial differences in urban basic public services, with inter-regional supervariable density contributing the most and becoming the main source of regional differences.

Third, from a correlation perspective, there is a significant positive relationship between the equalization of basic public services and the regional gap in economic resilience. Additionally, regression analysis also indicates that the equalization of basic public services has a significant impact on reducing the economic resilience gap across different regions. These findings suggest that equalizing basic public services plays a crucial role in narrowing the economic resilience gap in different regions. Therefore, it is necessary and feasible to coordinate the improvement of economic resilience in various regions by prioritizing equalization of basic public services. It is worth noting that these results remain robust even when measured by GDP per capita.

Based on the above findings, in order to promote the equalization of basic public services in China and improve economic resilience, this paper puts forward the following policy recommendations.

First, it is necessary to establish and improve the adjustment mechanism of the financial system to ensure that residents enjoy public services fairly. First, we should optimize the structure of fiscal expenditure and increase the proportion of fiscal expenditure on basic public services. Targeted support will increase the scale of fiscal spending on social security, medical and health care, education, infrastructure and employment security, and improve the level of public service supply; Second, promote the democratization and scientificization of the formulation and implementation of public service policies. The Administrative Procedure Law, the administrative accountability system, the rational cadre evaluation system, and the hearing system are powerful measures to ensure the scientific formulation and implementation of public service policies. Finally, increase the supply of institutions. At present, the level of market-oriented allocation of factors in China is low, and the existence of policies and systems such as household registration system, land system, and urban-rural dual system may be an obstacle to the equalization of basic public services in China. In addition, it is necessary to pay attention to improving the public’s ability to enjoy basic public services, and at the same time improve the public’s ability to choose public services, and increase the purchasing power of public services for groups in difficulty.

Second, we need to equalize basic public services in accordance with local conditions to enhance economic resilience. The results show that there is an unequal phenomenon in the supply of basic public services in China, and regional differences are the main source of differences in the level of basic public service supply, so different measures need to be taken according to the resource endowment of different regions to promote the equalization of basic public services. On the one hand, it is necessary to continue to implement the strategy of coordinated regional development and improve the financial capacity of local governments in backward areas by influencing the pattern of primary distribution. By intensifying support for the development of the central and western regions, we can focus on implementing industrial policies that are compatible with local resource endowments, achieve sound and sustainable economic development, and provide sufficient financial resources for the supply of basic public services. On the other hand, it is necessary to increase the "hematopoietic" transfer payment to underdeveloped areas, and narrow the financial gap between local governments in different regions based on secondary distribution.

Third, for developing countries, compulsory education and health care should be the focus of promoting the equalization of public services. Compulsory education and medical and health care are important basic public services related to the development of people’s livelihood and society, which have an important impact on the improvement of residents’ development potential and the reduction of expenditure costs. However, there are great differences in the quantity and quality of the provision of compulsory education and basic medical and health services in developing countries, so firstly, it is necessary to optimize the overall coordination mechanism of the government, improve the funding guarantee system, improve the supervision and evaluation system, and improve the top-level design of basic public education and medical and health services. secondly, we should scientifically allocate teacher resources, implement facilities and standards, promote the exchange and sharing of resources, and promote the comprehensive compliance of basic public education and medical and health services. Ensure service opportunities for the floating population, optimize and improve urban and rural planning, and improve the efficiency of the supply of basic public education and medical and health services.

We need to pay attention to the comprehensive impact of multiple factors and comprehensively promote the regional synergy of economic resilience. We must unswervingly continue to promote the innovation, coordination, openness, and efficiency of economic development. To achieve this, we should improve the technological innovation ability of enterprises, strengthen the main position of enterprise innovation, promote the deep integration of production, education, and research, and give play to the entrepreneurial spirit. The leading and supporting role of large enterprises should be utilized, while learning from foreign ways and methods to encourage independent innovation, improve China’s key core technologies, and "stuck neck" technology. We should solve the social and people’s livelihood problems caused by "stuck neck" technology and improve the structure of R&D investment. Property rights should be protected, and the level of achievement transformation should be improved. The proportion of endogenous R&D investment of enterprises should be increased to enhance the innovation ability of enterprises.

It is important to note that China’s 2014 intellectual property index ranked only 47th among 97 countries, and the level of intellectual property protection was in a low position. The lack of property rights would reduce enterprise innovation. We should improve the transformation of innovation achievements and reduce the output of waste patents. Furthermore, improving total factor productivity, especially green total factor productivity, is crucial. We should adhere to the folio method, not only focusing on "quantity" but also improving "quality". We should also promote the coordination of industrial structure, urban and rural structure, and regional structure development. The current industrial structure in China is unreasonable, and although the proportion of added value of the tertiary industry to GDP increased to 54.5% by 2020, there is still a long way to go from the 70% of the tertiary industry in developed countries. Therefore, the tertiary industry should be actively developed. The dual structure of urban and rural areas is a serious issue, with the urban-rural income ratio reaching 2.56, while the urban-rural income ratio of developed countries is about 1.5. Although each country’s national conditions are different, China’s urban-rural structure is obviously unreasonable. It is necessary to actively stabilize the countryside, serve farmers, and develop modern agriculture to increase farmers’ income, improve their living consumption level, and narrow the urban-rural gap. Finally, we should expand the scale of credit of financial institutions and tilt credit to private enterprises and small and medium-sized enterprises. This will help private enterprises and small and medium-sized enterprises to get rid of the dilemma of financing difficulties.

## Supporting information

S1 Data (XLSX)
